# Altered Amino Acid Metabolism in Patients with Cardiorenal Syndrome Type 2: Is It a Problem for Protein and Exercise Prescriptions?

**DOI:** 10.3390/nu13051632

**Published:** 2021-05-13

**Authors:** Roberto Aquilani, Roberto Maestri, Maurizia Dossena, Maria Teresa La Rovere, Daniela Buonocore, Federica Boschi, Manuela Verri

**Affiliations:** 1 Department of Biology and Biotechnology “Lazzaro Spallanzani”, University of Pavia, 27100 Pavia, Italy; dottore.aquilani@gmail.com (R.A.); maurizia.dossena@unipv.it (M.D.); daniela.buonocore@unipv.it (D.B.); 2 Department of Biomedical Engineering of the Montescano Institute, Istituti Clinici Scientifici Maugeri IRCCS, 27040 Montescano, Italy; roberto.maestri@icsmaugeri.it; 3 Department of Cardiac Rehabilitation of the Montescano Institute, Istituti Clinici Scientifici Maugeri IRCCS, 27040 Montescano, Italy; mariateresa.larovere@icsmaugeri.it; 4 Department of Drug Sciences, University of Pavia, 27100 Pavia, Italy; federica.boschi@unipv.it

**Keywords:** cardiorenal syndrome, plasma amino acids, multiorgan impact, practical implications

## Abstract

The goal of this retrospective study was to document any alterations in plasma amino acids (AAs) in subjects with cardiorenal syndrome type 2 (CRS 2). We analyzed data from sixteen patients with CRS 2 and eight healthy subjects (control group, C), whose plasma arterial (A) and venous (V) AA concentrations had been measured. Compared to C, the group of CRS 2 patients showed significant reductions by more than 90% in A (*p* < 0.01) and V (*p* < 0.01) individual AAs, whereas negative A-V differences that indicated a net muscle AA release (muscle hypercatabolism) were found in 59% of CRS 2 patients (*p* < 0.03). No significant differences in plasma A and V AA concentrations nor in A-V differences were found between patients with mild kidney damage (*N* = 5; estimated glomerular filtration rate, eGFR ≥ 60 mL/min/1.73 m^2^) and patients with moderate-severe kidney damage (*N* = 11; eGFR < 60 mL/min/1.73 m^2^). Several plasma arterial AAs correlated with hemodynamic variables, but not with GFR. The study showed that patients with CRS 2 had very low concentrations of circulating AAs, independent of the degree of GFR damage.

## 1. Introduction

The complication of chronic heart failure (CHF) with chronic kidney disease (CKD) identifies the cardiorenal syndrome (CRS) which is classified as CRS type 2 (CRS 2) [1,2]. The prevalence of CRS 2 is estimated to be 25–63% [3,4,5]. The development of CKD in the CHF setting amplifies the clinical difficulties in managing volume overload, using mechanical circulatory support in a cardiac transplantation [6]. Anemia, cachexia and physical deconditioning, which are three independent risk factors of survival and functional prognosis in CHF [7,8,9], might be aggravated. The development of renal failure reduces survival, even in patients with preserved left ventricular ejection fraction (LVEF) [3].

We hypothesized that the development of renal dysfunction in patients with CHF may amplify the alterations of amino acid (AA)/protein metabolism, as reflected by plasma AA concentrations, already documented in CHF alone [10] and in primary CKD alone [11]. Firstly, CHF and CKD share similar pathogenic mechanisms, influencing the turnover of body/muscle nitrogen metabolism. These mechanisms include hemodynamic factors such as right ventricular overload [1,12], neurohormonal activation with sympathetic overdrive, activation of renin-angiotensin-aldosterone system (RAAS), inflammation, hormonal alterations and immune dysregulation [1,13]. Secondly, the kidney plays a major role on body AA homeostasis [14,15]. Thirdly, in CRS 2, both glomerular [16] and tubular [2,17,18,19] damages are described.

The presence of an abnormal plasma amino acid (PAA) profile in patients with CRS 2 may be clinically important given that it has the potential to impair the metabolic activities of all body districts, including the heart and the kidney themselves, thus acting as additive damage. Moreover, abnormal PAAs may lead the physician to two therapeutic dilemmas: (a) when patients are stable, how could their dietary protein intake be reduced in relation to glomerular filtration rate (GFR) damage [20] and, at the same time, how could it be increased in order to correct abnormal PAAs and provide patients with an adequate amount of nitrogen for their body’s metabolic requirements? (b) During an acute event, would normal or artificial nutrition be adequate to support the body’s increased nitrogen needs?

Using the data from a previous study where we had analyzed arterial and venous PAA profile in CHF patients [10], we performed a secondary analysis on the collected data. The aim of the analysis was to investigate whether GFR damage could be associated with abnormal PAA concentrations, even though we were aware that proximal tubules are the main structure deputed to AA reabsorption from filtered plasma.

## 2. Materials and Methods

We re-analyzed the data from chronic heart failure (CHF) patients who had participated in a previous study on Plasma Amino Acid Abnormalities [10]. These patients were admitted to the Heart Failure Unit of the Scientific Institute of Montescano to undergo right cardiac catheterization for heart transplantation evaluation. We only selected CRS 2 patients whose arterial and venous AAs had been measured after overnight fasting.

The diagnosis of CRS 2 was established following the indication of the American Heart Association Statement [1]. In addition to PAA measurements, the inclusion criteria were the following: clinical stability (no changes in drugs over the previous three weeks, and no clinical evidence of body water retention), stable normal body weight (body mass index, BMI, > 22 kg/m^2^) for the previous three months, absence of hypoglycemic agents, normal liver function (total bilirubin < 1.1 mg/dL; serum alanine aminotransferase < 39 U/L; serum oxaloacetic aminotransferase < 25 U/L), absence of kidney dysfunction preceding the diagnosis of CHF, absence of primary endocrine disturbances.

Following the routine protocol of the Institute, 2D-echocardiography and cardiopulmonary exercise testing was performed on CHF patients, and their venous N-terminal pro-B-type natriuretic peptide (NT-pro-BNP) concentrations were measured.

In the selected patients, CKD was diagnosed after transforming serum creatinine concentrations into GFR (estimated GFR, eGFR) (mL/min/1.73 m^2^) [21]. The eGFR values were then categorized according to the classification of Kidney Disease: Improving Global Outcomes (KDIGO) [22]. According to KDIGO, we identified 16 patients with CRS 2: 11 (68.8%) had eGFR < 60 mL/min/1.73 m^2^ (moderate-severe CKD: MS-CKD) (range 59–26) and 5 (31.2%) had eGFR ≥ 60 mL/min/1.73 m^2^ (range 60–88) (mild reduction: M-CKD). The patient characteristics have been reported in Table 1.

In the patients, the PAAs had been determined as described elsewhere [23], and expressed in µmol/L.

We used arterial (A) and venous (V) concentrations to calculate the AA (A-V) differences. A positive value indicated net muscle AA uptake (prevalence of anabolic activity); a negative value indicated a net muscle AA release (prevalence of catabolic activity); no positive–no negative (A-V) value indicated no AA net uptake, no net release (balanced muscle AA metabolism).

PAA concentrations were determined in a group of healthy subjects (controls: C; *N* = 8, 6 of whom males). The controls were selected for similar BMI (27.1 ± 2.2 kg/m^2^) and age (51 ± 9 years) and for absence of discretionary physical activity. The healthy subjects reported no significant past medical history. Examination of the control subjects confirmed that they were in good health. In healthy C, in the current study, we also considered the AA ornithine, which had not been considered in the previous study [10], as this AA is a by-product of the urea cycle; however, we did not consider the AA taurine [10] because it was not available in CRS 2 patients’ venous blood samples.

### Statistical Analysis

The central tendency and dispersion of continuous variables were reported as mean ± SD. Due to violations to the normality assumption (Shapiro–Wilk statistic), hypothesis testing was based on non-parametric statistics. Descriptive statistics for categorical variables were reported as N (percent frequency). Between-group comparisons were carried out by the Mann–Whitney U-test (two groups), or by the Kruskal–Wallis test (three groups) and by the Chi-square test for continuous and categorical variables, respectively. When the Kruskal–Wallis test was significant, post hoc analysis was carried out (Dunn–Sidak adjustment). The association between couples of variables was assessed by the Spearman’s correlation coefficient.

A *p*-value < 0.05 was considered statistically significant. All analyses were carried out using the SAS/STAT statistical package, release 9.4 (SAS Institute Inc., Cary, NC, USA).

## 3. Results

### 3.1. Comparison between Healthy Controls and the Entire Population with CRS 2

The study found significant differences in PAAs between the entire population with CRS 2 and C. In CRS 2, more than 90% of both arterial (Table 2) and venous (Table 3) individual AAs, and 71.4% of (A-V) differences (Table 4) were lower than in C. In CRS 2, total arterial and venous AAs (TAAs) were lower: −73% and −56.4%, respectively. In contrast, the muscle release of TAAs was higher (+453%, *p* = 0.027) in CRS 2 than in C. Moreover, in CRS 2, significantly lower arterial and venous essential amino acid (EAA) and branched chain amino acid (BCAA) concentrations were found (*p* = 0.0001 for all AAs), whereas their muscle releases (Table 4) were higher than in C. Compared to C, CRS 2 had arterial/venous AA ratios < 1 (Table 5).

To sum up, the study found that in comparison to controls, patients with CRS 2 had low PAAs even though their skeletal muscle tissue released a larger amount of these substrates.

### 3.2. Comparisons between C, M-CKD, MS-CKD

Compared to C, M-CKD patients (eGFR ≥ 60 mL/min/1.73 m^2^) had lower concentrations of arterial (Table 6) and venous (Table 7) TAAs, EAAs, BCAAs and all the single AAs, with the exception of venous threonine, which was similar in C and M-CKD. Skeletal muscle tissue in M-CKD (Table 8) released larger amounts of leucine, BCAAs and EAAs.

Compared to C, MS-CKD (eGFR < 60 mL/min/1.73 m^2^) patients had lower arterial (Table 6) and venous (Table 7) concentrations of all individual AAs, except for venous ornithine, which was higher. With respect to muscle AA (A-V) differences (Table 8), MS-CKD released larger amounts of leucine, EAAs, BCAAs, glycine, tyrosine, tryptophan and phenylalanine.

M-CKD and MS-CKD patients had similar concentrations of arterial and venous AAs as well as muscle AA releases.

### 3.3. Comparison of Non-Amino Acid Variables between M-CKD and MS-CKD Patients

Table 9 shows that the two subgroups of CKD had similar concentrations of all the variables, with the exception of creatinine, which was lower in M-CKD than in MS-CKD patients.

### 3.4. Correlations between Arterial Plasma AAs, Renal and Cardiac Functions

eGFR was positively associated with arterial systolic and diastolic blood pressures (*r* = +0.51, *p* = 0.055 and *r* = 0.72, *p* = 0.002, respectively). Several plasma AAs correlated with LVEF and other hemodynamic variables, but not with eGFR (Table 10).

## 4. Discussion

The study found that patients with CRS 2 had low arterial and venous PAA concentrations, even though these metabolic substrates were excessively released by skeletal muscle tissue. The rate of PAA deterioration was independent of the renal filtration damage.

PAA deterioration was worse in CHF and CKD patients when the two diseases were considered together than when they were considered separately. When CHF patients were considered alone, only aspartic acid, methionine, taurine (NYHA II and III) and glutamic acid, and cysteine (NYHA III) were low [10]. Notably, in patients with M-CKD, altered PAAs were similar to those observed in the CHF IV NYHA class [10]. In the early stages of CKD (stage I and II), only valine and leucine concentrations were lower than in controls [24], whereas in severe CKD (GFR of 7 mL/min) [25] there were increases in several non-essential AAs and decreases in five essential AAs (threonine, tryptophan, histidine, valine, leucine). Notably, PAA alterations in CRS 2 patients were greater than those observed in long-term hemodialyzed patients in whom 70% of arterial AAs were altered [26]. Higher concentrations of ornithine in MS-CKD patients than in normal subjects suggests overactivity of the urea cycle.

The study results clearly indicate that it is clinically and metabolically important in every patient with CHF to convert the serum creatinine levels into eGFR.

The non-dependence of AA alterations on the degree of GFR reduction clearly indicates that tubular dysfunction contributes to altered PAA concentrations. Under physiological conditions, proximal tubule cells reabsorb 80% of the filtrated AAs [27]. In CHF, a tubulo-interstitial injury may coexist with normal glomerular filtration [28] and is more evident during acute decompensation of heart failure [17,19]. Urinary levels of tubular markers are increased in clinically stable CHF [28] and may indicate impaired GFR even before GFR reduction [28]. Therefore, tubular injuries may bring about increased urinary AA loss [2,29].

The complex interplay of several factors shared by CHF and CKD likely provides an explanation of the results of this study. These factors include neurohormonal activation [30], consisting of hyperactivities of hypothalamic-pituitary-adrenal axis, adrenergic system, renin-angiotensin-aldosterone system (RAAS), hormonal imbalances [11,31,32], systemic inflammation [13,25,30,33,34], body/muscle AA overconsumption [10] and abnormal skeletal muscle intermediate metabolism [30,35,36,37]. All these factors alter body hemodynamic and rheologic conditions, and metabolic homeostasis and lead to changes in body AA/protein metabolism.

The main contributions of these factors to abnormal PAAs in CRS will be discussed separately for arterial AA concentrations and A-V differences.

### 4.1. Potential Mechanisms Underlying Low Arterial AAs

AA concentrations in arterial plasma reflect body protein metabolism better than venous plasma [38].

Under physiological conditions, arterial AA concentrations depend on both dietary protein intake and body protein metabolism [39].

Due to the lack of information about patients’ nutritional intakes, the role of nutrition in deteriorating PAAs cannot be delineated. However, it would be reasonable to assume that even if the patients’ nutrition had been normal, it would have been inadequate to meet the body’s nitrogen requirements, as inferred by the reduction in circulating essential AAs (EAAs): substances which must be provided by exogenous sources.

The combination of hemodynamic alterations, body AA overconsumption and intracellular metabolic acidosis (not determined in the study patients) may be responsible for the altered PAAs.

Both in CHF and CKD, volume overload leads to intestinal wall congestion [40,41], thus favoring the development of pathogenic gut flora [40,42]. Intestinal dysbiosis, in turn, may decrease the retrieval of non-absorbed protein [43] and, at the same time, may induce endoluminal proteolytic over-activity and urea formation. In addition, intestinal edema and gut dysbiosis are directly responsible for translocation of bacteria and/or their toxic products into the blood stream [42], causing/enhancing systemic inflammation. Low circulating citrulline suggests that the study patients may have had a dysfunctional small bowel mucosa. This amino acid is not incorporated in proteins, and is almost exclusively formed by enterocytes [44] and 80% of its concentration is converted into arginine in proximal convoluted kidney tubules [45]. Therefore, low citrulline reflects low intestinal production and/or increased intestinal ureagenesis.

The heart, the lungs, the kidney and skeletal muscle are body districts with high AA consumption. In heart failure, there is AA overconsumption to sustain myocardium remodeling, a process requiring a high rate of protein synthesis and oxidative metabolism [10,46,47]. Renal dysfunction itself increases the heart remodeling rate, given that on one hand renal disfunction (eGFR 60 mL/min/1.73 m^2^) is associated with left ventricular remodeling [48] and on the other hand, the accompanying increase in extracellular water induces left ventricular hypertrophy at a very early stage of chronic kidney disease [48].

In CHF, there is also AA overconsumption in the lungs [49], in particular in subjects without β-blocker therapy [50]. Renal dysfunction per se causes AA overconsumption due to gluconeogenesis, ureagenesis and structural remodeling because of tubular hypertrophy that is caused by the concentration of ammonia in the tubule cells [51].

Metabolic acidosis lowers PAA concentrations by increasing renal AA uptake and, at the same time, suppressing renal proteolysis [52]. Measures of acid-base balance were not available in the study patients, however intracellular metabolic acidosis could be suspected given the low arterial histidine concentration, an important intracellular buffer [53].

Skeletal muscle AA utilization, particularly in mitochondria, occurs both in CHF and CKD as documented in bioptic specimens from the quadriceps muscle of CHF [35] and CKD [36], showing exalted mitochondrial aminotransferase activities.

At first glance, low arterial AAs could be due to low venous AA concentrations; however, this is not a major mechanism as the patients, unlike controls, had low arterial/venous AA ratios, indicating that muscle release of AAs, although in excess, was not enough to balance AA uptake by extramuscular body districts.

In summary, the study suggests that in CRS 2 patients, the body’s AA requirements are greater than the amount of AAs provided by the skeletal muscle, which is the main store of AAs in the body.

### 4.2. Muscle AA (A-V) Differences and AA Plasma Venous Concentrations

The net muscle AA releases, in particular phenylalanine, indirectly indicate the presence of muscle protein hypercatabolism [54], whose pathophysiological mechanisms are shared by both CHF and CKD, and include inflammation [30,33,55,56] and hemodynamic factors such as venous congestion and hypertension, metabolic acidosis, insulin, and growth hormone resistances.

Inflammation is a potent condition causing muscle AA depletion. The overproduction of proinflammatory cytokines increases muscle protein degradation, inhibits both muscle protein synthesis and repair [57,58,59,60,61,62,63] and increases production of catabolic hormones such as glucagon, catecholamines and cortisol [64,65].

Both in CHF and CKD, one source of cytokine production is venous hypertension [66].

In CKD, metabolic acidosis leads to muscle AA overconsumption by accelerating protein degradation [67].

Insulin and growth hormone (GH) resistances reduce anabolic activities both in CHF [68] and CKD [11,69] as insulin resistance depresses the antiproteolytic activity of the insulin [70], in particular during overnight fasting, and GH resistance causes muscle proteolysis given that the physiologic GH activity is to increase AA uptake into skeletal muscle.

### 4.3. Correlations between Cardiac Function, Renal Function and PAAs in CRS 2

The study confirms the positive correlations existing between most PAAs and cardiac function [10]. On the contrary, no significant correlations were found between PAAs and renal filtration rate (GFR), suggesting that renal glomeruli have no role in body AA metabolic homeostasis.

Regarding the heart/kidney relationship, the positive association between renal filtration rate and arterial blood pressure (both systolic and diastolic pressures) indicates that an important determinant of GFR is the peripheral arterial pressure and consequently the renal perfusion pressure [71] and not the cardiac output as also documented by a previous study [72].

The lack of information about patient nutritional intake does not allow us to understand the contribution of ingested salt and water intakes to the hyponatremia found in MS-CKD. Given that sodium is the most important osmotic solute of extracellular fluid, it would be reasonable to assume that serum osmolarity was low in MS-CKD, and low concentrations of circulating AAs likely contributed to reduce osmolarity. Notably, increased blood glucose and urea may help to maintain renal perfusion pressure, mitigate sodium-induced hypoosmolality, and limit the extravasation of intravascular hypotonic fluid towards the intracellular and interstitial spaces.

The higher serum albumin in MS-CKD could be due to higher protein-calorie intakes [73] and/or a hypovolemic state associated with hypotonic hyponatremia. This latter mechanism may be plausible given that the patients were not on hypertonic infusions, nor did they have serious hyperglycemia, hyperlipidemia or hyperproteinemia.

The similar PAA alterations in M-CKD and MS-CKD indicate that the renal contribution to altered AA/protein metabolism starts in the early stages of renal damage in subjects with CHF.

### 4.4. Relevance of Altered PAAs for Patients with CRS 2. Potential Practical Implications

In CRS 2 patients, the alterations of AA/protein metabolism may potentially contribute to and accentuate the metabolic and functional alterations of several body districts (Table 11).

The hypoaminoacidemia in hyperazotemic CRS 2 patients raises the question of whether it is appropriate to prescribe a hypoproteic diet before improving circulating AAs. The authors’ opinion is that a hypoproteic diet should be prescribed in association with EAA supplementation for the following reasons. Firstly, a hypoproteic diet alone may further impair circulating AAs. Secondly, a bolus of oral 8 g EAAs has shown to increase EAA plasma concentrations in healthy subjects [81]. Chronic EAA supplementation has been shown to improve body weight, anthropometric measures, insulin resistance and exercise tolerance in stable CHF on rehabilitative treatment [82,83]. Interestingly, in CKD patients, physical exercise can improve protein energy wasting [84,85,86]. Thirdly, 8 g free EAAs, by providing 1.28 g nitrogen vs. 3.28 g nitrogen from 100 g lean beef meat with a similar quantity of EAAs, can save nitrogen and at the same time ensure/enhance anabolic activities. Lastly, CHF patients release a large amount of AAs during light exercise that mimics the physical activities of daily life [87]. Future research should address whether the association of EAA supplementation and physical training could benefit hyperazotemic CRS 2 in terms of improvements in the PAA profile and body/muscle anabolic activity.

It would be prudent to improve plasma AA concentrations when the patients are clinically stable in order to limit metabolic damage following periods of acute events requiring continuous renal replacement therapy or during hemodynamic instability [88].

This study suggests the importance of calculating nutritional intakes of patients with CHF as soon as CHF is first diagnosed, given the high prevalence of the development of renal damage.

### 4.5. Limitations of the Study

The study has several limitations that should be addressed by future research. The results of the study should be confirmed by a prospective investigation with a larger patient population.

Patients’ nutritional intakes and body tissue composition were not available. The knowledge of nutritional intakes would have allowed us to better understand the contribution of diet to circulating AAs. Body composition analysis would have allowed us to diagnose a state of sarcopenia or cachexia. However, a depletion of skeletal muscle mass in the study patients may be likely, given their muscle hypercatabolism [89].

A population of subjects with CHF alone was not considered in this study. For comparison aims, we referred to the abnormal plasma AA profile of subjects with CHF described in a previous investigation [10]. Similarly, the study did not compare AA concentrations in CRS 2 patients with those in CKD patients alone.

Renal biomarkers [90] including plasma beta 2-microglobulin [91], N-acetyl-beta-glucosaminidase (NAG) [92] and urinary kidney injury molecule-1 (KIM-1) [93] were not available in this study. Thus, a prospective investigation is necessary to address the relationship between levels of kidney injury markers and plasma AA levels.

Another limitation of the study is the lack of information about urine AA losses. This information would have strengthened the discussion. A future prospective study will address the balance between urine and plasma amino acid levels.

The knowledge of patients’ acid-base state would have allowed us to better understand its contribution to muscle net AA releases. In addition, the determination of urinary AA losses would have suggested the role played by proximal tubular dysfunction in contributing to altered PAAs.

## 5. Conclusions

The study shows that patients with cardiorenal syndrome type 2 had very low concentrations of circulating AAs, the rates of which were independent of the degree of GFR reduction.

## Figures and Tables

**Table 1:** Demographic, anthropometric and clinical characteristics, functional class, etiology, biohumoral variables, cardiac hemodynamic variables and renal function tests of the studied cardiorenal syndrome type 2 (CRS 2) patients.

Variables	All-CRS 2 (*N* = 16)
*Demographics*	
Age (years)	56.5 ± 8.5
Sex (male/female)	11/5
*Anthropometrics*	
Body weight (kg)	76.0 ± 15.2
BMI (kg/m^2^)	26.3 ± 3.9
*Blood*	
Glucose (mg/dL; NV = 80–110)	96.3 ± 14.1
Albumin (g/dL; NV = 3.5–5)	4.3 ± 0.4
Hemoglobin (g/dL; NV = 12–15)	13.0 ± 2.2
Sodium (mEq/L; NV = 135–145)	136.4 ± 3.5
Potassium (mEq/L; NV = 3.5–5.0)	4.1 ± 0.6
NT-pro-BNP	2940.9 ± 1865.4
(pg/mL; NV < 125 for age < 75 years)	
*Clinical characteristics*	
*Medication*	
β blockers	16 pts (100%)
Diuretics	16 pts (100%)
ACE inhibition	14 pts (87.5%)
Digoxin	7 pts (43.7%)
*Functional class*	
NYHA	3.2 ± 0.5
*Etiology*	
Ischemic	10 pts (62.5%)
Idiopathic dilated cardiomyopathy	4 pts (25%)
Valvular	2 pts (12.5%)
*Arterial blood pressure*	
Systolic blood pressure (mm Hg)	108.4 ± 12.3
Diastolic blood pressure (mm Hg)	65.5 ± 11.4
*Hemodynamic variables*	
C_I_ (L/min/m^2^)	2.1 ± 0.4
SV (mL/beat)	61.5 ± 13.7
SV_I_ (mL/beat/m^2^)	32.8 ± 7.5
LVEF (%; NV > 55)	29.3 ± 12.0
*Physical performance*	
VO_2_ rest (mL O_2_/kg/min)	3.5 ± 0.8
VO_2_ peak (mL O_2_/kg/min)	11.8 ± 2.9
RER peak	1.10 ± 0.03
*Renal function tests*	
Creatinine (mg/dL; NV = 0.6–1.2)	1.42 ± 0.18
eGFR (mL/min/1.73 m^2^)	54.9 ± 19.3
Urea (mg/dL; NV = 20–40)	68.2 ± 46.2

Data are given as mean ± SD, except for gender. Abbreviations: CRS 2, cardiorenal syndrome type 2; BMI, body mass index; NT-pro-BNP, N-terminal pro-B-type natriuretic peptide; NYHA, New York Heart Association; C_I_, cardiac index; SV, stroke volume; SV_I_, stroke volume index; LVEF, left ventricular ejection fraction; NV, normal value; VO_2_, oxygen consumption; RER, respiratory exchange ratio; eGFR, estimated glomerular filtration rate.

**Table 2:** Plasma arterial AA concentrations (µmol/L) in controls (C) and cardiorenal syndrome type 2 (CRS 2) patients.

Variable	C (*N* = 8)	CRS 2 (*N* = 16)	*p*-Value
Aspartic acid	112.1 ± 8.858	29.83 ± 14.23	<0.0001
Glutamic acid	198.63 ± 10.61	84.97 ± 26.72	<0.0001
Asparagine	61.04 ± 1.99	23.60 ± 11.17	<0.0001
Serine	88.39 ± 4.25	23.17 ± 8.01	<0.0001
Glutamine	464.88 ± 13.98	100.64 ± 43.78	<0.0001
Histidine	58.00 ± 5.15	23.02 ± 33.44	0.014
Glycine	268.25 ± 11.97	49.08 ± 16.79	<0.0001
Threonine	111.6 ± 7.3	20.62 ± 10.00	<0.0001
Citrulline	24.57 ± 3.66	6.42 ± 1.73	<0.0001
Alanine	312.63 ± 15.67	72.28 ± 20.20	<0.0001
Arginine	59.27 ± 7.61	27.23 ± 11.36	0.00019
Tyrosine	56.25 ± 6.11	16.12 ± 3.90	<0.0001
Cysteine	77.13 ± 5.14	15.47 ± 6.37	<0.0001
Valine	160.0 ± 15.8	49.84 ± 12.79	<0.0001
Methionine	9.7 ± 2.8	3.22 ± 1.49	<0.0001
Tryptophan	50.1 ± 4.9	15.64 ± 7.93	<0.0001
Phenylalanine	51.3 ± 5.1	11.61 ± 3.17	<0.0001
Isoleucine	47.4 ± 4.1	10.61 ± 3.35	<0.0001
Leucine	79.1 ± 8.5	21.03 ± 6.93	<0.0001
Lysine	107 ± 11.4	37.65 ± 12.95	<0.0001
Ornithine	56.38 ± 6.39	63.88 ± 20.73	0.36
TAAs	2453.78 ± 49.54	626.56 ± 176.22	<0.0001
BCAAs	286.5 ± 13.57	81.47 ± 22.41	<0.0001
EAAs	612.2 ± 20.3	170.21 ± 48.64	<0.0001

Data are given as mean ± SD. Reported *p*-values are from Mann–Whitney U-test. AA, amino acid; C, controls; CRS 2, cardiorenal syndrome type 2; TAAs, total amino acids; BCAAs, branched chain amino acids; EAAs, essential amino acids.

**Table 3:** Plasma venous AA concentrations (µmol/L) in controls (C) and cardiorenal syndrome type 2 (CRS 2) patients.

Variable	C (*N* = 8)	CRS 2 (*N* = 16)	*p*-Value
Aspartic acid	111.83 ± 10.48	43.48 ± 15.53	<0.0001
Glutamic acid	206.13 ± 11.15	154.01 ± 52.32	0.004
Asparagine	112.65 ± 157.39	41.27 ± 10.38	0.001
Serine	90.83 ± 4.21	42.10 ± 14.69	<0.0001
Glutamine	467.25 ± 11.67	128.17 ± 31.67	<0.0001
Histidine	58.38 ± 6.02	37.92 ± 27.40	0.043
Glycine	258.38 ± 27.54	90.67 ± 38.11	<0.0001
Threonine	106.63 ± 11.10	28.83 ± 8.42	<0.0001
Citrulline	25.07 ± 2.90	9.77 ± 3.26	<0.0001
Alanine	327.63 ± 15.60	125.82 ± 37.67	<0.0001
Arginine	59.44 ± 5.85	36.21 ± 16.75	0.001
Tyrosine	51.75 ± 5.70	36.04 ± 12.97	0.003
Cysteine	79.38 ± 7.76	26.00 ± 27.58	0.0006
Valine	153.88 ± 13.48	79.44 ± 26.50	<0.0001
Methionine	10.75 ± 1.75	6.61 ± 3.47	0.003
Tryptophan	51.13 ± 4.64	35.09 ± 15.89	0.003
Phenylalanine	46.25 ± 5.68	22.99 ± 7.37	<0.0001
Isoleucine	45.75 ± 5.01	19.27 ± 5.27	<0.0001
Leucine	78.13 ± 6.36	43.50 ± 12.10	<0.0001
Lysine	115.75 ± 11.03	59.02 ± 17.97	<0.0001
Ornithine	55.38 ± 6.72	98.37 ± 30.20	0.0009
TAAs	2377.56 ± 151.78	1040.19 ± 210.78	<0.0001
BCAAs	277.75 ± 12.96	142.21 ± 42.71	<0.0001
EAAs	608.25 ± 19.95	294.75 ± 71.74	<0.0001

Data are given as mean ± SD. Reported *p*-values are from Mann–Whitney U-test. AA, amino acid; C, controls; CRS 2, cardiorenal syndrome type 2; TAAs, total amino acids; BCAAs, branched chain amino acids; EAAs, essential amino acids.

**Table 4:** (A-V) AA differences (µmol/L) in controls (C) and cardiorenal syndrome type 2 (CRS 2) patients.

Variable	(A-V) C (*N* = 8)	(A-V) CRS 2 (*N* = 16)	*p*-Value
Aspartic acid	0.27 ± 14.35	−13.65 ± 20.73	0.10
Glutamic acid	−7.50 ± 21.05	−69.04 ± 70.60	0.027
Asparagine	−51.61 ± 157.21	−17.67 ± 16.03	0.023
Serine	−2.44 ± 6.45	−18.93 ± 18.59	0.032
Glutamine	−2.38 ± 23.05	−27.53 ± 54.47	0.08
Histidine	−0.38 ± 5.04	−14.90 ± 48.43	0.023
Glycine	9.88 ± 29.98	−41.60 ± 32.73	0.001
Threonine	4.97 ± 14.84	−8.21 ± 10.40	0.11
Citrulline	−0.50 ± 5.65	−3.35 ± 2.67	0.14
Alanine	−15.00 ± 20.54	−53.53 ± 38.40	0.012
Arginine	−0.16 ± 10.21	−8.98 ± 14.61	0.14
Tyrosine	4.50 ± 9.10	−19.92 ± 13.23	0.0006
Cysteine	−2.25 ± 8.10	−10.53 ± 27.13	0.95
Valine	6.12 ± 19.39	−29.60 ± 25.15	0.017
Methionine	−1.05 ± 1.93	−3.39 ± 4.45	0.037
Tryptophan	−1.03 ± 7.65	−19.45 ± 18.86	0.008
Phenylalanine	5.05 ± 6.56	−11.38 ± 7.94	0.006
Isoleucine	1.65 ± 5.72	−8.66 ± 5.96	0.023
Leucine	0.97 ± 7.25	−22.47 ± 14.06	0.002
Lysine	−8.75 ± 16.73	−21.37 ± 21.05	0.020
Ornithine	1.00 ± 10.49	−34.50 ± 32.73	0.004
TAAs	76.22 ± 138.9	−413.63 ± 333.21	0.020
BCAAs	8.75 ± 23.87	−60.74 ± 43.95	0.006
EAAs	7.95 ± 24.07	−124.54 ± 92.02	0.004

Data are given as mean ± SD. Reported *p*-values are from Mann–Whitney U-test. AA, amino acid; A, arterial AA concentration; V, venous AA concentration; C, controls; TAAs, total amino acids; BCAAs, branched chain amino acids; EAAs, essential amino acids.

**Table 5:** Plasma arterial/venous ratios (%) in controls (C) and cardiorenal syndrome type 2 (CRS 2) patients.

Variable	Ratio C (*N* = 8)	Ratio CRS 2 (*N* = 16)	*p*-Value
Aspartic acid	1.00 ± 0.13	0.77 ± 0.48	0.09
Glutamic acid	0.97 ± 0.10	0.87 ± 1.22	0.027
Asparagine	0.96 ± 0.35	0.62 ± 0.33	0.032
Serine	0.98 ± 0.07	0.63 ± 0.33	0.017
Glutamine	1.00 ± 0.05	0.84 ± 0.44	0.10
Histidine	1.00 ± 0.09	0.61 ± 0.11	0.014
Glycine	1.05 ± 0.13	0.97 ± 1.71	0.0006
Threonine	1.05 ± 0.14	0.75 ± 0.37	0.014
Citrulline	1.00 ± 0.24	0.70 ± 0.21	0.010
Alanine	0.96 ± 0.06	0.61 ± 0.23	0.003
Arginine	1.01 ± 0.17	0.83 ± 0.34	0.09
Tyrosine	1.10 ± 0.17	0.53 ± 0.33	0.002
Cysteine	0.98 ± 0.11	0.93 ± 0.45	0.54
Valine	1.04 ± 0.12	0.68 ± 0.26	0.008
Methionine	0.90 ± 0.17	0.78 ± 0.90	0.017
Tryptophan	0.98 ± 0.15	0.56 ± 0.47	0.020
Phenylalanine	1.11 ± 0.15	0.56 ± 0.28	0.002
Isoleucine	1.04 ± 0.13	0.59 ± 0.26	0.002
Leucine	1.01 ± 0.09	0.53 ± 0.28	0.002
Lysine	0.92 ± 0.14	0.68 ± 0.28	0.007
Ornithine	1.03 ± 0.19	0.70 ± 0.27	0.007
TAAs	1.03 ± 0.06	0.65 ± 0.29	0.008
BCAAs	1.03 ± 0.09	0.62 ± 0.26	0.003
EAAs	1.00 ± 0.04	0.62 ± 0.28	0.003

Data are given as mean ± SD. Reported *p*-values are from Mann–Whitney U-test. C, controls; CRS 2, cardiorenal syndrome type 2; TAAs, total amino acids; BCAAs, branched chain amino acids; EAAs, essential amino acids.

**Table 6:** Plasma arterial AA concentrations (µmol/L) in controls (C) and cardiorenal syndrome type 2 (CRS 2) patients after stratification for eGFR ≥ 60 mL/min/1.73 m^2^ (mild CKD: M-CKD) and eGFR < 60 mL/min/1.73 m^2^ (moderate-severe CKD: MS-CKD).

Variable	C (*N* = 8)	M-CKD eGFR ≥ 60 mL/min/1.73 m^2^ (*N* = 5)	MS-CKD eGFR < 60 mL/min/1.73 m^2^ (*N* = 11)	*p*-Value	*p*-Value C vs. M-CKD	*p*-Value C vs. MS-CKD	*p*-Value M-CKD vs. MS-CKD
Aspartic acid	112.1 ± 8.858	30.49 ± 19.76	29.53 ± 12.13	0.00046	0.009	0.0007	1.00
Glutamic acid	198.63 ± 10.61	80.48 ± 29.08	87.01 ± 26.80	0.00045	0.005	0.001	0.99
Asparagine	61.04 ± 1.99	20.21 ± 9.20	25.13 ± 12.04	0.00040	0.003	0.002	0.94
Serine	88.39 ± 4.25	21.80 ± 8.97	23.79 ± 7.92	0.00046	0.007	0.0009	1.00
Glutamine	464.88 ± 13.98	100.16 ± 33.41	100.86 ± 49.28	0.00045	0.013	0.0006	1.00
Histidine	58.00 ± 5.15	21.52 ± 32.02	23.71 ± 35.57	0.050	0.18	0.066	1.00
Glycine	268.25 ± 11.97	47.53 ± 18.66	49.78 ± 16.79	0.00046	0.009	0.0007	1.00
Threonine	111.6 ± 7.3	21.63 ± 8.88	20.16 ± 10.85	0.00045	0.015	0.0005	0.99
Citrulline	24.57 ± 3.66	5.98 ± 2.48	6.62 ± 1.37	0.00044	0.004	0.001	0.98
Alanine	312.63 ± 15.67	67.30 ± 7.58	74.55 ± 23.89	0.00046	0.007	0.0009	1.00
Arginine	59.27 ± 7.61	28.52 ± 17.02	26.64 ± 8.75	0.0009	0.011	0.002	1.00
Tyrosine	56.25 ± 6.11	14.81 ± 3.74	16.72 ± 3.99	0.00035	0.002	0.002	0.85
Cysteine	77.13 ± 5.14	12.73 ± 6.32	16.72 ± 6.27	0.00037	0.002	0.002	0.89
Valine	160.0 ± 15.8	47.73 ± 12.95	50.80 ± 13.24	0.00044	0.005	0.001	0.99
Methionine	9.7 ± 2.8	3.12 ± 1.27	3.26 ± 1.64	0.00043	0.004	0.001	0.98
Tryptophan	50.1 ± 4.9	17.44 ± 11.53	14.82 ± 6.22	0.00046	0.008	0.0008	1.00
Phenylalanine	51.3 ± 5.1	9.99 ± 2.41	12.35 ± 3.30	0.00033	0.001	0.002	0.81
Isoleucine	47.4 ± 4.1	10.41 ± 2.53	10.70 ± 3.78	0.00045	0.006	0.001	1.00
Leucine	79.1 ± 8.5	19.98 ± 6.28	21.50 ± 7.45	0.00043	0.004	0.001	0.98
Lysine	107 ± 11.4	32.37 ± 7.34	40.05 ± 14.48	0.00037	0.002	0.002	0.89
Ornithine	56.38 ± 6.39	60.34 ± 28.54	65.48 ± 17.59	0.43	-	-	-
TAAs	2453.78 ± 49.54	601.46 ± 166.72	637.97 ± 187.10	0.00046	0.007	0.0009	1.00
BCAAs	286.5 ± 13.57	78.12 ± 20.75	83.00 ± 23.94	0.00045	0.005	0.001	0.99
EAAs	612.2 ± 20.3	162.67 ± 45.03	173.63 ± 51.92	0.00046	0.007	0.0009	1.00

Data are given as mean ± SD. Reported *p*-values are from Kruskal–Wallis test. Post hoc *p*-values (Dunn–Sidak adjustment) are reported for the following comparisons: controls vs. mild chronic kidney disease (C vs. M-CKD), controls vs. moderate-severe chronic kidney disease (C vs. MS-CKD) and mild chronic kidney disease vs. moderate-severe chronic kidney disease (M-CKD vs. MS-CKD). AA, amino acid; C, controls; CRS 2, cardiorenal syndrome type 2; eGFR, estimated glomerular filtration rate; CKD, chronic kidney disease; M-CKD, mild CKD; MS-CKD, moderate-severe CKD; TAAs, total amino acids; BCAAs, branched chain amino acids; EAAs, essential amino acids.

**Table 7:** Plasma venous AA concentrations (µmol/L) in controls (C) and cardiorenal syndrome type 2 (CRS 2) patients after stratification for eGFR ≥ 60 mL/min/1.73 m^2^ (mild CKD: M-CKD) and eGFR < 60 mL/min/1.73 m^2^ (moderate-severe CKD: MS-CKD).

Variable	C (*N* = 8)	M-CKD eGFR ≥ 60 mL/min/1.73 m^2^ (*N* = 5)	MS-CKD eGFR < 60 mL/min/1.73 m^2^ (*N* = 11)	*p*-Value	*p*-Value C vs. M-CKD	*p*-Value C vs. MS-CKD	*p*-Value M-CKD vs. MS-CKD
Aspartic acid	111.83 ± 10.48	36.54 ± 15.10	46.63 ± 15.35	0.00029	0.0010	0.003	0.71
Glutamic acid	206.13 ± 11.15	155.13 ± 34.08	153.49 ± 60.34	0.014	0.034	0.037	0.94
Asparagine	112.65 ± 157.39	39.04 ± 11.23	42.2 8 ± 10.37	0.005	0.017	0.013	0.96
Serine	90.83 ± 4.21	43.43 ± 20.09	41.49 ± 12.69	0.00045	0.006	0.001	1.00
Glutamine	467.25 ± 11.67	130.22 ± 28.59	127.24 ± 34.27	0.00044	0.015	0.0005	0.99
Histidine	58.38 ± 6.02	38.37 ± 27.63	37.72 ± 28.64	0.12	-	-	-
Glycine	258.38 ± 27.54	96.28 ± 51.79	88.13 ± 32.90	0.00044	0.018	0.00048	0.98
Threonine	106.63 ± 11.10	33.64 ± 6.98	26.64 ± 8.37	0.00031	0.048	0.00021	0.76
Citrulline	25.07 ± 2.90	8.27 ± 3.65	10.45 ± 2.99	0.00034	0.001	0.002	0.81
Alanine	327.63 ± 15.60	126.17 ± 48.59	125.65 ± 34.40	0.00044	0.005	0.001	0.99
Arginine	59.44 ± 5.85	32.47 ± 8.91	37.91 ± 19.47	0.005	0.017	0.013	0.96
Tyrosine	51.75 ± 5.70	32.30 ± 9.96	37.74 ± 14.23	0.008	0.014	0.038	0.78
Cysteine	79.38 ± 7.76	11.81 ± 5.83	32.45 ± 31.32	0.0010	0.001	0.023	0.38
Valine	153.88 ± 13.48	78.79 ± 32.25	79.74 ± 25.25	0.00046	0.007	0.0009	1.00
Methionine	10.75 ± 1.75	6.45 ± 3.29	6.68 ± 3.70	0.013	0.062	0.021	1.00
Tryptophan	51.13 ± 4.64	31.57 ± 10.19	36.69 ± 18.12	0.013	0.042	0.027	0.98
Phenylalanine	46.25 ± 5.68	20.59 ± 8.91	24.08 ± 6.75	0.00040	0.003	0.002	0.94
Isoleucine	45.75 ± 5.01	19.25 ± 6.31	19.28 ± 5.07	0.00046	0.007	0.0009	1.00
Leucine	78.13 ± 6.36	43.58 ± 13.99	43.46 ± 11.89	0.00046	0.009	0.0007	1.00
Lysine	115.75 ± 11.03	62.10 ± 23.14	57.62 ± 16.23	0.00046	0.009	0.0007	1.00
Ornithine	55.38 ± 6.72	99.14 ± 35.47	98.02 ± 29.40	0.004	0.028	0.007	1.00
TAAs	2377.56 ± 151.78	1034.19 ± 248.63	1042.92 ± 204.67	0.00046	0.008	0.0008	1.00
BCAAs	277.75 ± 12.96	141.62 ± 51.16	142.48 ± 41.10	0.00045	0.006	0.001	1.00
EAAs	608.25 ± 19.95	295.97 ± 86.42	294.19 ± 68.79	0.00046	0.008	0.0008	1.00

Data are given as mean ± SD. Reported *p*-values are from Kruskal–Wallis test. Post hoc *p*-values (Dunn–Sidak adjustment) are reported for the following comparisons: controls vs. mild chronic kidney disease (C vs. M-CKD), controls vs. moderate-severe chronic kidney disease (C vs. MS-CKD) and mild chronic kidney disease vs. moderate-severe chronic kidney disease (M-CKD vs. MS-CKD). AA, amino acid; C, controls; CRS 2, cardiorenal syndrome type 2; eGFR, estimated glomerular filtration rate; CKD, chronic kidney disease; M-CKD, mild CKD; MS-CKD, moderate-severe CKD; TAAs, total amino acids; BCAAs, branched chain amino acids; EAAs, essential amino acids.

**Table 8:** (A-V) AA differences (µmol/L) in controls (C) and cardiorenal syndrome type 2 (CRS 2) patients after stratification for eGFR ≥ 60 mL/min/1.73 m^2^ (mild CKD: M-CKD) and eGFR < 60 mL/min/1.73 m^2^ (moderate-severe CKD: MS-CKD).

Variable	C (*N* = 8)	M-CKD eGFR ≥ 60 mL/min/1.73 m^2^ (*N* = 5)	MS-CKD eGFR < 60 mL/min/1.73 m^2^ (*N*=11)	*p*-Value	*p*-Value C vs. M-CKD	*p*-Value C vs. MS-CKD	*p*-Value M-CKD vs. MS-CKD
Aspartic acid	0.27 ± 14.35	−6.05 ± 21.62	−17.10 ± 20.38	0.14	-	-	-
Glutamic acid	−7.50 ± 21.05	−74.65 ± 56.04	−66.48 ±7 8.73	0.09	-	-	-
Asparagine	−51.61 ± 157.21	−18.83 ± 11.79	−17.14 ± 18.13	0.067	-	-	-
Serine	−2.44 ± 6.45	−21.63 ± 24.06	−17.71 ± 16.77	0.10	-	-	-
Glutamine	−2.38 ± 23.05	−30.06 ± 53.39	−26.38 ± 57.49	0.19	-	-	-
Histidine	−0.38 ± 5.04	−16.86 ± 29.60	−14.01 ± 56.25	0.057	-	-	-
Glycine	9.88 ± 29.98	−48.74 ± 45.88	−38.35 ± 26.97	0.006	0.051	0.008	1.00
Threonine	4.97 ± 14.84	−12.01 ± 9.50	−6.48 ± 10.76	0.19	-	-	-
Citrulline	−0.50 ± 5.65	−2.30 ± 1.98	−3.83 ± 2.89	0.22	-	-	-
Alanine	−15.00 ± 20.54	−58.87 ± 51.47	−51.10 ± 33.64	0.042	0.13	0.064	1.00
Arginine	−0.16 ± 10.21	−3.94 ± 16.48	−11.27 ± 13.90	0.25	-	-	-
Tyrosine	4.50 ± 9.10	−17.49 ± 13.04	−21.02 ± 13.80	0.003	0.054	0.003	0.97
Cysteine	−2.25 ± 8.10	0.92 ± 4.04	−15.73 ± 31.66	0.56	-	-	-
Valine	6.12 ± 19.39	−31.06 ± 25.46	−28.94 ± 26.23	0.057	-	-	-
Methionine	−1.05 ± 1.93	−3.33 ± 4.43	−3.42 ± 4.68	0.11	-	-	-
Tryptophan	−1.03 ± 7.65	−14.13 ± 17.42	−21.87 ± 19.78	0.026	0.29	0.022	0.91
Phenylalanine	5.05 ± 6.56	−10.60 ± 7.40	−11.73 ± 8.50	0.022	0.16	0.023	0.99
Isoleucine	1.65 ± 5.72	−8.84 ± 6.88	−8.58 ± 5.86	0.08	-	-	-
Leucine	0.97 ± 7.25	−23.60 ± 16.03	−21.96 ± 13.88	0.009	0.030	0.019	0.98
Lysine	−8.75 ± 16.73	−29.74 ± 26.63	−17.57 ± 18.17	0.056	-	-	-
Ornithine	1.00 ± 10.49	−38.80 ± 44.59	−32.54 ± 28.25	0.016	0.10	0.020	1.00
TAAs	76.22 ± 138.9	−432.73 ± 384.01	−404.95 ± 327.56	0.065	-	-	-
BCAAs	8.75 ± 23.87	−63.50 ± 47.20	−59.48 ± 44.73	0.022	0.08	0.037	1.00
EAAs	7.95 ± 24.07	−133.30 ± 100.00	−120.56 ± 92.98	0.014	0.034	0.037	0.94

Data are given as mean ± SD. Reported *p*-values are from Kruskal–Wallis test. Post hoc *p*-values (Dunn–Sidak adjustment) are reported for the following comparisons: controls vs. mild chronic kidney disease (C vs. M-CKD), controls vs. moderate-severe chronic kidney disease (C vs. MS-CKD) and mild chronic kidney disease vs. moderate-severe chronic kidney disease (M-CKD vs. MS-CKD). AA, amino acid; A, arterial AA concentration; V, venous AA concentration; C, controls; CRS 2, cardiorenal syndrome type 2; eGFR, estimated glomerular filtration rate; CKD, chronic kidney disease; M-CKD, mild CKD; MS-CKD, moderate-severe CKD; TAAs, total amino acids; BCAAs, branched chain amino acids; EAAs, essential amino acids.

**Table 9:** Blood non-amino acid variables in mild CKD (M-CKD; eGFR ≥ 60 mL/min/1.73 m^2^) and moderate-severe CKD (MS-CKD; eGFR < 60 mL/min/1.73 m^2^) patients.

Variable	M-CKD eGFR ≥ 60 mL/min/1.73 m^2^ (*N* = 5)	MS-CKD eGFR < 60 mL/min/1.73 m^2^ (*N* = 11)	*p*-Values
Serum osmolarity (mOsm/L; NV = 290–320)	271.36 ± 5.35	277.08 ± 16.38	0.78
Plasma bilirubin (mg/dL; NV = 0.5–1.0)	0.80 ± 0.34	1.10 ± 0.53	0.28
Serum sodium (mEq/L; NV = 135–145)	137.80 ± 3.11	135.82 ± 3.63	0.31
Serum creatinine (mg/dL; NV = 0.6–1.2)	1.02 ± 0.08	1.60 ± 0.44	0.002
Serum potassium (mEq/L; NV = 3.5–5.0)	4.22 ± 0.59	4.10 ± 0.58	0.95
Plasma urea (mg/dL; NV = 20–40)	50.80 ± 24.45	76.09 ± 52.39	0.26
Plasma glucose (mg/dL; NV = 80–110)	93.20 ± 10.13	97.73 ± 15.84	0.61
Blood hemoglobin (g/dL; NV = 12–15)	13.34 ± 2.53	12.93 ± 2.18	0.67
Serum albumin (g/dL; NV = 3.5–5)	4.07 ± 0.58	4.54 ± 0.31	0.08
Total cholesterol (mg/dL; NV < 200)	146.40 ± 22.39	162.09 ± 53.35	0.61
Plasma triglycerides (mg/dL; NV = 60–170)	98.40 ± 33.40	112.90 ± 71.05	1.00

Data are given as mean ± SD. Reported *p*-values are from Mann–Whitney U-test. CKD, chronic kidney disease; M-CKD, mild CKD; MS-CKD, moderate-severe CKD; eGFR, estimated glomerular filtration rate; NV, normal value.

**Table 10:** Correlation coefficients (Spearman’s *r*) between plasma arterial concentrations of individual AAs (µmol/L), and renal function and hemodynamic variables.

	eGFR (mL/min/1.73 m^2^)	RAP (mmHg)	LVEF (%)	LVEDD (mm)	LVESD (mm)	C_I_ (L/min/m^2^)
Aspartic acid	0.18	−0.03	0.53 ^	0.23	0.21	0.56 ^
Glutamic acid	−0.04	−0.08	0.42	0.32	0.29	0.51
Asparagine	−0.11	0.46	0.49	−0.03	−0.04	0.14
Serine	0.02	−0.01	0.26	0.06	−0.05	0.57 ^
Glutamine	0.28	−0.04	0.44	0.38	0.28	0.58 ^
Histidine	−0.03	−0.14	0.16	0.42	0.34	0.12
Glycine	−0.23	−0.12	−0.37	0.05	0.04	−0.06
Threonine	0.20	−0.33	0.35	0.24	0.14	0.18
Citrulline	−0.29	−0.61 ^	−0.09	0.40	0.37	0.17
Alanine	−0.21	−0.51	−0.03	0.59 ^	0.49	0.30
Arginine	−0.25	0.03	0.21	0.03	0.02	0.40
Tyrosine	−0.14	−0.06	0.57 ^	−0.10	−0.02	0.21
Cysteine	−0.17	−0.2	0.22	−0.21	−0.09	−0.06
Valine	0.17	−0.30	0.57 ^	0.11	0.02	0.22
Methionine	−0.09	−0.17	0.19	−0.15	−0.11	−0.12
Tryptophan	−0.01	−0.17	0.22	0.40	0.38	0.62 ^
Phenylalanine	−0.13	−0.06	0.51 ^	0.04	−0.10	0.29
Isoleucine	0.28	−0.20	0.64 †	0.09	0.04	0.41
Leucine	0.15	−0.41	0.61 ^	0.07	0.06	0.34
Lysine	−0.13	−0.05	0.50 ^	0.05	0.08	0.19
Ornithine	−0.30	0.22	−0.15	0.12	−0.09	−0.11

^: *p* < 0.05; †: *p* < 0.01; AA, amino acid; eGFR, estimated glomerular filtration rate; RAP, right atrial pressure; LVEF, left ventricular ejection fraction; LVEDD, left ventricular end-diastolic diameter; LVESD, left ventricular end-systolic diameter; C_I_, cardiac index.

**Table 11:** Some examples of potential additive damage to altered physiology of CRS 2 patients from hypoaminoacidemia.

Metabolic Compartments	Effects	Metabolic and Clinical Impacts
Protein synthesis		
(a) visceral compartment	reduced albumin synthesis [73] reduced erythropoietin synthesis [74] reduced immune cell proliferation, differentiation, function [75,76]	hypoalbuminemia anemia impaired immune response
(b) somatic compartment (skeletal muscle tissue)	reduced contractile myofibrils [77]	sarcopenia, reduced muscle strength
Brain	decreased fuel provision decreased neurotransmitter synthesis [78]	altered cognition, behavior, mood, appetite
Intestine metabolism	reduced energy metabolism reduced protein synthesis [79]	small intestine injury: mucosal barrier disruption bacteria/toxins translocation
Kidney metabolism	reduced renal mTOR complex signaling [27]	increased tubuli mitochondria dysfunction impaired mitochondria biogenesis reduced protein synthesis reduced nucleotide synthesis increased oxidative stress
Heart metabolism	mitochondrial dysfunction altered myocardium remodeling increased oxidative stress [10]	inadequate energy production maladaptive remodeling reduced left ventricular ejection fraction
Lung metabolism	reduced activity of alveolar Na^+^/K^+^ pump [50]	accumulation of intralveolar fluid
Acid-base balance	reduced intracellular protein and AA buffers alterations in intermediate metabolism [80]	exaltation of intracellular acidosis reduced energy production increased oxidative stress

## Data Availability

Data supporting the reported results were confidential. The datasets that were used and/or analyzed during the current study, but not shown in the paper, are available from the corresponding author, on reasonable request.
